# Spotted Fever and Typhus Group Rickettsiae in Dogs and Humans, Mexico, 2022

**DOI:** 10.3201/eid2907.230333

**Published:** 2023-07

**Authors:** Ricardo Palacios-Santana, Lihua Wei, Nadia A. Fernandez-Santos, Mario A. Rodriguez-Perez, Sergio Uriegas-Camargo, Nicole L. Mendell, Donald H. Bouyer, Jose Guillermo Estrada-Franco

**Affiliations:** Instituto Politecnico Nacional, Centro de Biotecnologia Genomica Nacional, Reynosa, Mexico (R. Palacios-Santana, L. Wei, N.A. Fernandez-Santos, M.A. Rodriguez-Perez, J.G. Estrada-Franco);; Texas A&M University, College Station, Texas, USA (N.A. Fernandez-Santos);; Secretaria de Salud de Tamaulipas, Ciudad Victoria, Mexico (S. Uriegas-Camargo);; University of Texas Medical Branch, Galveston, Texas, USA (N.L. Mendell, D.H. Bouyer)

**Keywords:** rickettsiae, bacteria, spotted fever, typhus, dogs, zoonoses, *Rickettsia*, canine, Mexico

## Abstract

We found serologic evidence of spotted fever group *Rickettsia* in humans and dogs and typhus group *Rickettsia* in dogs in Reynosa, Mexico. Our investigation revealed serologic samples reactive to spotted fever group *Rickettsia* in 5 community members, which highlights a potential rickettsial transmission scenario in this region.

The spotted fever group (SFG) rickettsiae, which are transmitted primarily by tick bite to rodents, dogs, wild animals, and persons, comprise a phylogenetically discrete clade of the family Rickettsiacae, encompassing ≈30 species. SFG rickettsioses represent a major cause of febrile illness worldwide. This group includes *Rickettsia rickettsii*, the bacterium responsible for Rocky Mountain spotted fever (RMSF), a major pathogen of public health concern linked to severe hemorrhagic illness in humans. SFG rickettsiae are maintained through a tick–vertebrate host cycle; humans are incidental hosts (*1*). The arthropod-borne typhus group (TG) comprises *R. typhi*, the causative agent of murine or endemic typhus, and *R*. *prowazekii*, the pathogen responsible for epidemic typhus (*2*). *R. typhi* is sustained in enzootic cycles by small mammals, such as rats (*Rattus* spp.) and opossums (*Didelphis* spp.), and by ectoparasites such as the rat flea (*Xenopsylla cheopis*) and cat flea (*Ctenocephalides felis)* (*3*,*4*). Direct transmission of TG rickettsiae to humans is by cross-contamination between mucosal or skin abrasions and *Rickettsia* contained in the feces of either fleas (*R. typhi*) or human body lice (*R. prowazekii*) or simply by inhalation of contaminated dust (*4*). However, a sylvatic cycle of *R. prowazekii* in the United States has been associated with ectoparasites of flying squirrels (*Glaucomys volans*) (*5*). Clinical manifestations of TG rickettsioses start with sudden fever onset and other nonspecific symptoms, including severe headaches, myalgias, arthralgias, nausea, and vomiting.

During 2013–2022, a total of 2,232 RMSF cases were reported in 5 of 6 northern states of Mexico contiguous to the United States (*6*). Endemicity of TG rickettsiae in the southern counties of Texas in the Rio Grande transborder area of the United States (*4*) and seropositivity among blood donors from Mexico City (*7*) and Yucatán (*8*), Mexico, indicate a need to evaluate TG rickettsiae presence in this area. We report a cross-sectional serologic study for SFG and TG rickettsiae conducted in domestic dogs and humans in Reynosa, an urban city in northeastern Mexico. On the basis of previous research in this city (*1*), we hypothesized that dogs are sentinels of infectious disease circulation, as has been reported elsewhere for rickettsiae (*9*,*10*), and therefore are useful in surveillance approaches to monitor rickettsial disease in humans.

## The Study

We tested 106 dogs from 71 households in 6 peri-urban neighborhoods of Reynosa during March 13–July 4, 2022 (Figure). We collected signed, individual informed consents from canine owners and recorded data about the sampled dogs, which included vaccine history, gender, age, and breed. We administered rabies vaccine to unvaccinated dogs. We recruited all household members (n = 36) owning SFG- or TG-seropositive dogs, 16 of whom provided blood samples for further analysis. We collected and processed blood samples (≈3– to 5 mL, dog or human) according to previously reported methods (*1*). We centrifuged blood at 3000 × *g* for 10 min and stored serum samples −80° C until further processing. We tested serum samples for IgG against *R. amblyommatis*, *R. parkeri*, *R. rickettsii*, and *R. typhi* by indirect immunofluorescence assay as previously described (*11*). We recorded serum samples with reciprocal iIgG titers ≥64 or higher as positive and determined endpoint titer of those samples.

**Figure:**
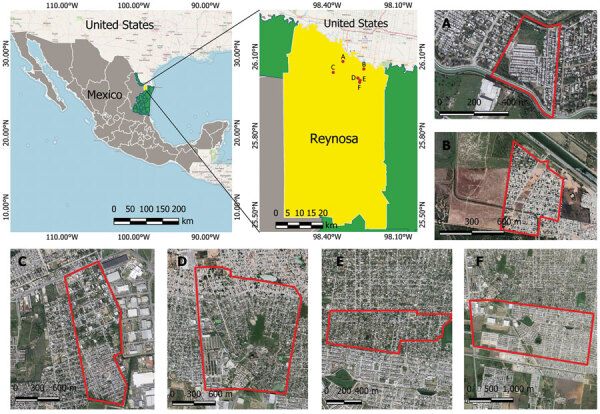
Sampling locations for study of spotted fever and typhus group rickettsiae in dogs and humans, in Reynosa, Mexico, 2022. Primary maps show location of Reynosa along the Mexico–United States border; neighborhoods sampled (A–F) are labeled and enlarged in the satellite images. Primary maps generated using QGIS 3.28.2 (https://www.qgis.org). Free geographic data of administrative areas of Mexico was downloaded from the National Institute of Statistics and Geography, Mexico (INEGI, https://www.inegi.org.mx/app/mapas). Satellite images and street maps were obtained from Google Maps (https://www.google.com/maps).

Of the dogs sampled, 5 (4.71%, 95% CI 0.68%–8.75%) were positive for SFG *Rickettsia* IgG and 4 (3.77%; 95% CI 0.15%–7.4%) were positive for TG *Rickettsia* IgG antibodies (Appendix Table 1). To further test whether seropositive dogs were sentinels of rickettsial exposure, we collected blood samples from dog owners and their families and questioned them about recent tick bites and clinical history (prior 3 months) associated with signs of rickettsial illness (Appendix Table 2) (*12*). Testing revealed that 5 (32.3%; 95% CI 1.84%–53.9%) of 16 serum samples obtained from owners in households with positive dogs were positive for SFG *Rickettsia* IgG using identical experimental conditions (Appendix Table 1). The Tamaulipas Health Services is conducting a follow-up investigation for the medical evaluation and treatment for the SFG-seropositive persons.

## Conclusion

RMSF was described in Mexico in 1943. We report finding antibodies to SFG *Rickettsia* in Reynosa, Mexico, in 2022. Seroevidence of domestic dog exposure to *R. typhi* in Reynosa warrants further investigation, especially given the endemicity of murine typhus in the contiguous Hidalgo County, Texas, USA, that involved >1,000 human cases reported during 2008–2019. Our results validate the theory that dogs can be sentinels for human diseases and demonstrated their assistance in inferring the temporal and spatial dynamics of some diseases in regions associated with their vectors (*12*).

Metropolitan Reynosa is an industrial center and a reference transborder point area of migration between different demographic groups comprising the Reynosa–Texas–US region, known as Rio Grande Valley. It is a land strip of ≈110 km housing ≈3 million permanent residents, with a large floating population fluctuating in the hundreds to thousands. The entire Mexico–United States transborder region spans 3,141 km, and >286 million crossings are documented annually (*13*). 

Historic outbreaks of RMSF along the Mexico–United States border region have been documented, with fatality rates of 30%–80% (*12*). In 2019, an RMSF seroprevalence of 6% (95% CI 4.68%−7.46%; 69/1,136 cases) in dogs was reported from 14 Mexico border cities in the states of Baja California, Sonora, and Coahuila (*14*), which is concordant with the seroprevalence of our study (4.71%; 95% CI 0.68%–8.75%). Human cases of murine typhus have been reported mainly in southeastern Mexico, but rarely in northeastern Mexico. However, endemicity in the Rio Grande Valley has been reported widely on the US side of the border (*4*). In 2022, 23.9% (95% CI 16.9%–31.0%; 34/142 samples) of human serum samples were positive for *R. typhi* IgG in Yucatan, and 15% of those were positive for *R. rickettsii* (*15*). Similarly, first screening of seropositive *Rickettsia* IgG in dogs informed identification of putative human cases, indicating this surveillance strategy is effective (*15*).

Most dogs roam freely in relatively squalid conditions throughout our studied areas. In this context, evidence of TG *Rickettsia* antibodies in dogs did not correspond to the same findings in their owners. On the contrary, SFG-seropositive owners of SFG-seronegative dogs suggests the possibility that the owners may have acquired the infection away from their residence and pets. Although antibody titers of 3 of 5 SFG-reactive human samples suggest *R. parkeri* as the presumptive agent, without paired serum samples, other SFG rickettsiae cannot be excluded. Because of rickettsial cross-reactivity observed by immunofluorescence assay, further studies using cross-absorption techniques to clarify the responsible etiologic agent(s) are warranted. This transborder transmission scenario appears to repeat along Mexico towns contiguous to the US border, where brown dog tick (*Rhipicephalus sanguineus*) infestations are known to be rampant. 

Our findings suggest that the epidemiologic fabric of the region is strongly affected by the high incidence of SFG and TG rickettsioses. We hypothesize that improvement of the urban environment, using a One Health approach, along with integrated vector control management of ectoparasites (e.g., dog tick collars, tick trapping, acaricide spraying) would be of utmost importance in reducing the spread of rickettsial diseases in regions such as our study area. Evidence of TG rickettsia exposure of domestic dogs in this study highlights the need for further surveillance to determine the vector phenology and transmission cycle in the region. In conclusion, SFG and TG rickettsiae surveillance and control, by both standard and novel approaches, are urgently needed for areas along the northeastern Mexico–United States border.

Appendix More information for study of spotted fever and typhus group rickettsiae in dogs and humans, Mexico, 2022.
