# Supplementary material for: Spotted Fever and Typhus Group Rickettsiae in Dogs and Humans, Mexico, 2022
Source: Emerg Infect Dis. 2023 Jul;29(7):1443–6. doi: 10.3201/eid2907.230333 (PMC10310375; doi:10.3201/eid2907.230333)
Supplement: Appendix — More information for study of spotted fever and typhus group rickettsiae in dogs and humans, Mexico, 2022. [file 23-0333-Techapp-s1.pdf]

Article DOI: http://doi.org/10.3201/eid2907.230333

EID cannot ensure accessibility for supplementary materials supplied by authors. Readers who have difficulty accessing supplementary content should contact the authors for assistance.

Spotted Fever and Typhus Group Rickettsiae in Dogs and Humans, Mexico, 2022

Appendix

Appendix Table 1. Reciprocal endpoint titer from dog and human serum samples for antibodies to spotted fever group or typhus group *Rickettsia*, Reynosa, Mexico, 2022*

				Dog serum sample IFA test results						Human serum sample IFA test results				
Dog ID	Breed	Dog Sex	Dog Age, y	Endpoint titer *R. rickettsii*	Endpoint titer *R. amblyommatis*	Endpoint titer *R. parkeri*	Endpoint titer TGR	Human Age, y	Human Sex	Endpoint titer *R. rickettsii*	Endpoint titer *R. amblyommatis*	Endpoint titer *R. parkeri*	Endpoint titer TGR	Location
5	Chihuahua	M	1.2	4096	1024	1024	NA	53	F	2048	512	4096	NA	B
16	Mongrel	F	7	8192	4096	4096	NA	71	M	2048	1024	4096	NA	B
30	Mongrel	F	7	256	512	128	NA	NA	NA	NA	NA	NA	NA	B
31	Mongrel	M	9	1024	2048	4096	NA	NA	NA	NA	NA	NA	NA	B
53	Mongrel	M	3	512	1024	1024	NA	77	M	4096	4096	32768†	NA	D
37	Chihuahua	M	1	NA	NA	NA	16384	47	F	4096	1024	16384†	NA	B
57	Mongrel	F	4	NA	NA	NA	16384	42	F	4096	1024	16384†	NA	D
68	Mongrel	M	3	NA	NA	NA	32768	NA	NA	NA	NA	NA	NA	D
84	Mongrel	F	3	NA	NA	NA	512	NA	NA	NA	NA	NA	NA	D

*IFA, indirect immunofluorescence assay; NA, not applicable; TGR, typhus group rickettsiosis
†Presumptive etiologic agent

Appendix Table 2. Symptomology of study participants, Reynosa, Mexico, 2022*

	Human					
Dog ID	Sex	Age, y	Neighborhood	Recent Tick bite	Symptoms	Result
5	F	53	B	N	Fever, muscle aches	SFGR+
16	M	71	B	Y	Fever, rash, fatigue, muscle aches, joint pain	SFGR+
	F	72	B	N	Fever	NA
37	F	21	B	N	NA	NA
	F	47	B	N	Fever, muscle aches, joint pain	SFGR+
	F	23	B	N	NA	NA
53	M	77	D	Y	Fever, muscle aches	SFGR+
	F	38	D	N	NA	NA
	F	15	D	N	NA	NA
	F	12	D	N	Headache, fatigue	NA
	F	56	D	N	Fever, muscle aches	NA
57	F	42	D	N	Fever, headache, fatigue	SFGR+
68	F	26	D	N	Headache, fatigue	NA
	M	3	D	N	NA	NA
	M	6	D	N	NA	NA
	M	7	D	N	NA	NA

*NA, not applicable, SFGR+, positive for spotted fever group rickettsiosis
